# Penicillin G concentrations required for prophylaxis against Group A *Streptococcus* infection evaluated using a hollow fibre model and mathematical modelling

**DOI:** 10.1093/jac/dkac124

**Published:** 2022-04-26

**Authors:** Jessica R Tait, Timothy C Barnett, Kate E Rogers, Wee Leng Lee, Madhu Page-Sharp, Laurens Manning, Ben J Boyd, Jonathan R Carapetis, Roger L Nation, Cornelia B Landersdorfer

**Affiliations:** Drug Delivery, Disposition and Dynamics, Monash Institute of Pharmaceutical Sciences, Monash University, Parkville, Victoria, Australia; Wesfarmers Centre for Vaccines and Infectious Diseases, Telethon Kids Institute, Perth, Western Australia, Australia; Drug Delivery, Disposition and Dynamics, Monash Institute of Pharmaceutical Sciences, Monash University, Parkville, Victoria, Australia; Drug Delivery, Disposition and Dynamics, Monash Institute of Pharmaceutical Sciences, Monash University, Parkville, Victoria, Australia; Curtin Medical School, Curtin University, Bentley, Western Australia, Australia; Wesfarmers Centre for Vaccines and Infectious Diseases, Telethon Kids Institute, Perth, Western Australia, Australia; Faculty of Health and Medical Sciences, University of Western Australia, Perth, Western Australia, Australia; Drug Delivery, Disposition and Dynamics, Monash Institute of Pharmaceutical Sciences, Monash University, Parkville, Victoria, Australia; Wesfarmers Centre for Vaccines and Infectious Diseases, Telethon Kids Institute, Perth, Western Australia, Australia; Faculty of Health and Medical Sciences, University of Western Australia, Perth, Western Australia, Australia; Department of Infectious Diseases, Perth Children’s Hospital, Perth, Western Australia, Australia; Drug Delivery, Disposition and Dynamics, Monash Institute of Pharmaceutical Sciences, Monash University, Parkville, Victoria, Australia; Drug Delivery, Disposition and Dynamics, Monash Institute of Pharmaceutical Sciences, Monash University, Parkville, Victoria, Australia

## Abstract

**Background:**

Acute rheumatic fever (ARF), an autoimmune reaction to Group A *Streptococcus* (*Streptococcus pyogenes*; Strep A) infection, can cause rheumatic heart disease (RHD). New formulations of long-acting penicillins are being developed for secondary prophylaxis of ARF and RHD.

**Objectives:**

To evaluate the penicillin G concentrations required to suppress growth of Strep A.

**Methods:**

Broth microdilution MIC and MBC for Strep A strains M75^611024^, M1T1^5448^ and M18^MGAS8232^ were determined. All strains were studied in a hollow fibre model (initial inoculum 4 log_10_ cfu/mL). Constant penicillin G concentrations of 0.008, 0.016 and 0.05 mg/L were examined against all strains, plus 0.012 mg/L against M18^MGAS8232^. Viable counts were determined over 144 h. Subsequently, all penicillin G-treated cartridges were emptied, reinoculated with 5 log_10_ cfu/mL and counts determined over a further 144 h. Mathematical modelling was performed.

**Results:**

MIC and MBC were 0.008 mg/L for all strains; small subpopulations of M75^611024^ and M1T1^5448^, but not M18^MGAS8232^, grew at 1× MIC. Following the first inoculation, 0.008 mg/L achieved limited killing and/or stasis against M75^611024^ and M1T1^5448^, with subsequent growth to ∼6 log_10_ cfu/mL. Following both inocula, concentrations ≥0.016 mg/L suppressed M75^611024^ and M1T1^5448^ to <1 log_10_ cfu/mL from 6 h onwards with eradication. Concentrations ≥0.008 mg/L suppressed M18^MGAS8232^ to <1 log_10_ cfu/mL from 24 h onwards with eradication after both inoculations. Mathematical modelling well described all strains using a single set of parameter estimates, except for different maximum bacterial concentrations and proportions of bacteria growing at 1× MIC.

**Conclusions:**

In the absence of validated animal and human challenge models, the study provides guidance on penicillin G target concentrations for development of new penicillin formulations.

## Introduction

Infections due to Group A *Streptococcus* (*Streptococcus pyogenes*; Strep A), e.g. pharyngitis, can cause an autoimmune response, which leads to acute rheumatic fever (ARF).^1–3^ The major cause of morbidity and mortality from ARF is chronic cardiac valvular damage, known as rheumatic heart disease (RHD). RHD has been estimated to affect 40.5 million people globally and cause 306* *000 deaths annually, mainly in low- and middle-income countries and among indigenous populations in high-income countries.^4–6^ Subsequent Strep A infections can cause recurrent ARF and each recurrence can lead to the progression of RHD.^2^ Secondary prevention of Strep A infections is thus critically important.^7^

The recommended first-line management for secondary prophylaxis of ARF and RHD is deep intramuscular injection of benzathine penicillin G (BPG) every 28 (or in some cases every 21) days.^8,9^ From the intramuscular injection site, BPG is absorbed very slowly into the central circulation and hydrolysed to penicillin G. Because of the slow absorption and hydrolysis, BPG results in much lower, but more sustained, systemic penicillin G concentrations than other parenteral penicillins. However, significant issues with the current formulation and route of administration, including pain, duration and frequency, are often cited as reasons for poor adherence.^9–11^ Therefore, there is substantial interest in new formulations for subcutaneous administration, including long-acting BPG implants.^9,12,13^ Such formulations aim to provide sustained release of penicillin G over prolonged time periods and substantially reduce the frequency and pain associated with the current intramuscular BPG injections. Ideally the target dose and release rate of new formulations should be underpinned by knowledge of the minimum penicillin G concentration required to prevent Strep A pharyngitis. A common assumption is that maintenance of plasma penicillin G concentrations above 0.02 mg/L is required to prevent Strep A pharyngitis and recurrences of ARF.^12,14–16^ This is based on the widely accepted MIC of 0.02 mg/L of penicillin G for Strep A; however, is not tailored to the variations that may occur between individual strains. In the absence of a validated human challenge model for the clinical endpoints (pharyngitis and recurrent ARF) or suitable animal model for prophylaxis of Strep A oropharyngeal colonization,^17^ exploring the pharmacokinetic/pharmacodynamic link between penicillin G concentration and inhibition of Strep A growth under conditions designed to simulate secondary prophylaxis provides an alternative approach. Hollow fibre infection models (HFIMs) offer substantial advantages over traditional static-concentration time–kill (SCTK) studies, as they can accurately achieve target exposures, prevent antibiotic degradation and be conducted over prolonged time periods.

The underlying pharmacokinetic/pharmacodynamic relationship and the effect of different penicillin G concentrations on multiple Strep A strains have never been studied over several days in a state-of-the-art *in vitro* infection model. Here, we investigated a range of concentrations of penicillin G over 6 days against multiple low inocula of three Strep A strains, using an HFIM in a novel application by simulating prophylaxis. We also developed a mathematical model to describe the time course of viable counts for Strep A in the HFIM.

## Materials and methods

### Antibiotic, bacterial strains and susceptibility testing

Solutions of penicillin G (penicillin G sodium salt; Lot 059M4826V; Sigma–Aldrich, St Louis, MO, USA) were prepared in sterile Milli-Q^®^ water immediately before each experiment. Mueller–Hinton broth (MHB; Becton Dickinson & Co., Sparks, MD, USA) supplemented with 2.5% lysed horse blood (LHB) was used in all experiments. Defibrinated whole horse blood (Media Preparation Unit, Melbourne University) was lysed by dilution and centrifugation to generate LHB. Mueller–Hinton agar (Becton Dickinson & Co.) supplemented with 5% defibrinated horse blood was used to streak out bacterial isolates from frozen stocks. Todd Hewitt agar supplemented with 1% yeast (THYA) was used to determine bacterial counts. THYA was prepared using Todd Hewitt broth powder and granulated agar (both Becton Dickinson & Co.) and yeast extract granulated for microbiology (Merck, Darmstadt, Germany).

Three strains of Strep A were examined: M75^611024^, M1T1^5448^ and M18^MGAS8232^.^18–20^ Broth microdilution MICs and MBCs (>99.9% decrease in bacterial concentration at 24 h compared with initial inoculum) were determined using MHB+2.5% LHB.^21,22^ MICs were determined in triplicate and MBCs in duplicate.

### Dynamic HFIM

The HFIM used cellulosic cartridges (C3008; FiberCell Systems, Frederick, MD, USA) in a humidified incubator at 36°C.^23,24^ The system was incubated for multiple days after set-up and before inoculation to confirm the absence of any contamination. A growth control and constant concentrations of 0.008, 0.016 and 0.05 mg/L penicillin G were evaluated against all strains. In addition, 0.012 mg/L penicillin G was evaluated against M18^MGAS8232^. The studied concentrations were chosen based on the MICs and MBCs for the strains. The penicillin G-containing medium (MHB* *+* *2.5% LHB) was kept in the fridge to avoid thermal degradation and changed every 24 h. The medium was delivered to the central reservoir of the HFIM at 0.5 mL/min (Masterflex L/S Cartridge Pump 7519-06; Cole-Parmer). To simulate prophylaxis, the penicillin G concentrations in the system were at the required constant concentrations before inoculation of the cartridges. Bacterial suspensions in log growth phase were prepared, the OD was measured spectrophotometrically and suspensions were adjusted to 4 log_10_ cfu/mL and injected into the cartridges.^17^ Samples (1.0 mL) were collected aseptically from each cartridge at 0, 2, 6, 24, 29, 48, 72, 96, 120 and 144 h for viable counting. To reduce antibiotic carry-over, samples were twice centrifuged at 4000 **g** for 5 min, with the supernatant decanted and the pellet resuspended in pre-warmed sterile saline. Samples were then manually plated onto THYA (limit of counting 1.0 log_10_ cfu/mL). Agar plates were incubated at 36°C for 48 h and colonies counted manually. Repeats of plating and counting were performed for a subset of samples. Since no resistance of Strep A to penicillin G has been observed,^25^ viable counting on penicillin G-containing agar at multiples of the MIC was not performed. All penicillin G-treated cartridges were emptied at 144 h after the first inoculation and reinoculated with 5 log_10_ cfu/mL. The M18^MGAS8232^ growth control was continued throughout the whole study (288 h in total) to confirm viability of the bacteria in the cartridge during the prolonged period of time. Following the second inoculation, serial samples were collected over a further 144 h. For cartridges that were clear, the whole cartridge volume was concentrated and plated to check for eradication at 144 h following each inoculation.

Media samples (1 mL) were obtained at various timepoints throughout the study from the central reservoir outflow of the HFIM and immediately stored at −80°C until analysis for pharmacokinetic validation. Penicillin G was measured based on a validated LC-MS/MS assay^26^ with the following modifications. A triple quadrupole mass spectrometer LC-MS/MS-8060 (Shimadzu, Kyoto, Japan) was used for analysis. The sample (20 μL) was mixed with 1 mL of hexane/ethyl acetate (20:80). After centrifugation, 0.9 mL of supernatant was dried in a vacuum evaporator at room temperature for 40 min. The dried sample was reconstituted in 100 μL of methanol and further diluted with 100 μL of water. The injection volume was 10 μL. Calibration curves were constructed from 2 to 200 ng/mL and were linear, with *r*^2^ ≥0.99. The intra-day and inter-day variability for the concentrations 5–100 ng/mL ranged from 4.2% to 9.4%. The accuracy calculated from the quality control samples for the concentrations 5–100 ng/mL were within the analytical ranges (94%–115%), confirming the assay performance. The lower limit of quantification and lower limit of detection were 2 and 1 ng/mL, respectively.

### Mathematical modelling

The data from all penicillin G concentrations and controls and all three strains were modelled simultaneously. S-ADAPT software (version 1.57, importance sampling algorithm, pmethod = 4) facilitated by SADAPT-TRAN was used for modeling.^27,28^ A life-cycle growth model describing the underlying biological processes involved in bacterial growth and replication was incorporated in the mechanism-based mathematical model (MBM).^29,30^ For isolates M75^611024^ and M1T1^5448^, the final model included two bacterial subpopulations existing in two states (i.e. vegetative or state 1 and replication or state 2) with different susceptibilities to penicillin G. The subpopulations were cfu_HS_ (highly susceptible to penicillin G) and cfu_LS_ (growing at 1× the broth microdilution MIC, i.e. slightly less susceptible to penicillin G). Only one subpopulation (cfu_HS_) was required to describe isolate M18^MGAS8232^. Bacterial transition from state 1 to state 2 was described by the first-order growth rate constant (*k*_12_) and followed by a fast replication, with the replication rate constant (*k*_21_) fixed at 50 h^−1^.^29,31^ The inverse of *k*_12_ was defined by the mean generation time.^32,33^

For M75^611024^ and M1T1^5448^, the total bacterial population was defined as:

(1) $$\mathrm{cf}u_{\mathrm{ALL}}=\mathrm{cf}u_{\mathrm{HS}1}+\mathrm{cf}u_{\mathrm{HS}2}+\mathrm{cf}u_{\mathrm{LS}1}+\mathrm{cf}u_{\mathrm{LS}2}$$

For M18^MGAS8232^, the total bacterial population was defined as:

(2) $$\mathrm{cf}u_{\mathrm{ALL}}=\mathrm{cf}u_{\mathrm{HS}1}+\mathrm{cf}u_{\mathrm{HS}2}$$

Equations (3) to (6) describe the cfu_HS_ subpopulation in state 1 (cfu_HS1_) and state 2 (cfu_HS2_). The same equations were applied to the cfu_LS_ subpopulations for M75^611024^ and M1T1^5448^, except for a different estimate of IC_50, Rep_, reflecting the ability of this subpopulation to grow at the MIC.

(3) $$d(\mathrm{cf}u_{\mathrm{HS}1})/\mathrm{dt}=2\cdot \mathrm{PLAT}\cdot (1\text{–}\mathrm{REPinh})\cdot k_{21}\cdot \mathrm{cf}u_{\mathrm{HS}2}-k_{12}\cdot \mathrm{cf}u_{\mathrm{HS}1}$$

(4) $$d(\mathrm{cf}u_{\mathrm{HS}2})/\mathrm{dt}=\text{–}k_{21}\cdot \mathrm{cf}u_{\mathrm{HS}2}+k_{12}\cdot \mathrm{cf}u_{\mathrm{HS}1}$$

The plateau factor (PLAT) represented the probability of successful replication.^29^

(5) $$\mathrm{PLAT}=1\text{–}[\mathrm{cf}u_{\mathrm{ALL}}/(\mathrm{cf}u_{\mathrm{ALL}}+\mathrm{cf}u_{\max})]$$

At low viable counts, PLAT approaches 1, representing a 100% probability of successful replication (in the absence of penicillin G). As cfu_ALL_ approaches cfu_max_, PLAT approaches 0.5, representing a 50% probability of successful replication where bacteria continue to transition between states 1 and 2, but the total viable count is constant.

The inhibition of successful bacterial replication by penicillin G was represented by REPinh as defined in Equation (6), where *C*_PEN_ was the concentration of penicillin G in broth.

(6) $$\mathrm{REPinh}=(\mathrm{Ima}x_{\mathrm{REP}}\cdot C_{\mathrm{PEN}})/(C_{\mathrm{PEN}}+IC_{50,\mathrm{REP},\mathrm{HS}})$$

At REPinh of 0.5, net stasis of the bacterial population is achieved. REPinh >0.5 yields bacterial killing because of unsuccessful replication in more than 50% of the bacterial population. All parameters are defined in Table 1.

**Table 1.** Parameter estimates of the mathematical model for penicillin G

Parameter	Symbol (unit)	Population mean estimate (SE%)		
		M75^611024^	M1T1^5448^	M18^MGAS8232^
Bacterial growth and subpopulations				
Initial inoculum				
first inoculation	Log_10_cfu_0,1st_	3.97 (2.1)		
second inoculation	Log_10_cfu_0,2nd_	4.88^a^ (1.7)/6.13^b^ (2.3)/8.89^c^ (1.3)		
Maximum population size	Log_10_cfu_max_	8.40 (3.4)		9.05 (1.2)
Mean generation time	MGT (min)	51.7 (4.3)		
Log_10_ proportion of bacteria growing at MIC	Log_10,LS_	−2.32 (9.8)^d^	−1.29 (17.9)^d^	^e^
Inhibition of successful replication by penicillin G				
Maximum inhibition of successful replication	Imax_REP_	1.0 (fixed)		
Penicillin G concentration causing 50% of Imax_REP_				
highly susceptible subpopulation	IC_50,REP,HS_ (mg/L)	0.00026^f^		
subpopulation growing at MIC	IC_50,REP,LS_ (mg/L)	0.0094 (8.6)		^e^
Residual variability				
Standard deviation of residual error on log_10_ scale	SD_cfu_	0.72 (12.0)	0.50 (14.7)	0.20 (13.4)

a Second inoculation of cartridges that were clear at 6 days after the first inoculation.

b Second inoculation of cartridges that had regrowth after first inoculation (0.008 mg/L penicillin G).

c Growth control, continued from first inoculation (no bacteria added after 6 days).

d −0.33 (37.1% SE) following second inoculation of cartridges that had regrowth after first inoculation.

e Only one subpopulation was required to describe M18^MGAS8232^ and therefore no estimate for subpopulation growing at MIC.

f Fixed to the estimate from M18^MGAS8232^.

The between-curve variability for model parameters was assumed to be log-normally distributed. For parameters constrained between 0 and 1, the between-curve variability was logistically transformed. The observed viable counts were fitted by an additive residual error model on log_10_ scale. For viable counts less than 10 colonies per plate, a residual error model as previously described was employed.^24,29^ The most predictive final model was distinguished based on the goodness of fit, the biological plausibility of parameter estimates and the S-ADAPT objective function value (−1 × log likelihood).

## Results

Broth microdilution MIC and MBC of penicillin G were 0.008 mg/L for all three isolates. Despite the same MBC, differences across the isolates were observed in the bacterial counts determined from the MIC wells at 24 h. Bacterial counts of M75^611024^ and M1T1^5448^ declined in a concentration-dependent fashion from 1.95–2.32 log_10_ cfu/mL at 0.008 mg/L penicillin G to ≤1.0 log_10_ cfu/mL at 0.125 mg/L, with no bacteria detected at 0.25 mg/L penicillin G. In contrast, M18^MGAS8232^ counts were <1.0 log_10_ cfu/mL (i.e. below the limit of counting) at all penicillin G concentrations ≥0.008 mg/L.

**Figure 1.**
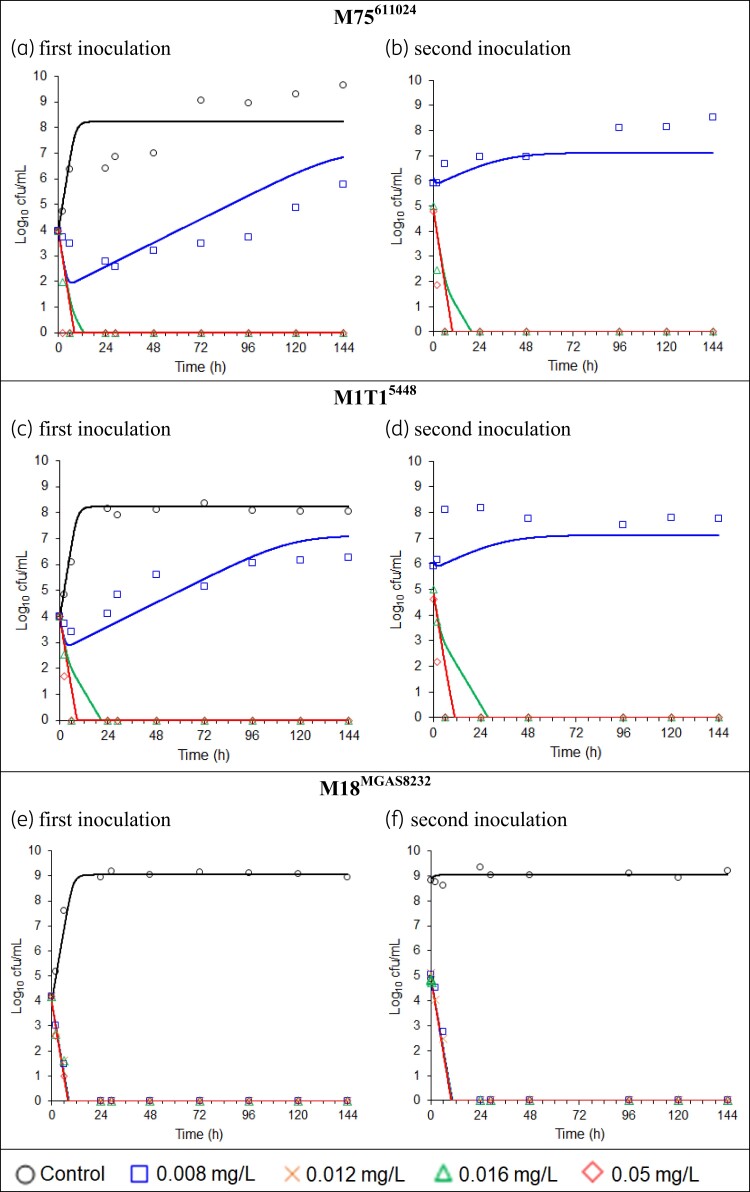
(a–f) Total viable counts and population fitted lines (without between-curve variability). The data from all penicillin G concentrations and controls and all three strains were modelled simultaneously. Samples below the limit of counting are plotted at zero. This figure appears in colour in the online version of *JAC* and in black and white in the print version of *JAC*.

The observed penicillin G concentrations in the HFIM were on average within 5.4% of the targeted concentrations. The viable count profiles over time of all three isolates, following the first and second inoculations, are shown in Figure 1. All growth controls plateaued at 8–9 log_10_ cfu/mL. The M18^MGAS8232^ growth control, which was continued for 12 days in total, remained stable at ∼9 log_10_ cfu/mL.

Following the first inoculation of the HFIM cartridge, penicillin G at 0.008 mg/L (1× the broth microdilution MIC) achieved limited bacterial killing and/or stasis against M75^611024^ and M1T1^5448^, followed by growth to ∼6 log_10_ cfu/mL at 144 h (Figure 1a and c). Due to the very viscous nature of the bacterial suspension at 144 h, not all bacteria could be removed from the cartridge and the bacterial counts at 0 h of the second inoculation were >5 log_10_ cfu/mL (Figure 1b and d). Virtually no effect of 0.008 mg/L penicillin G was observed after the second inoculation. In contrast, exposure to 1× the broth microdilution MIC suppressed M18^MGAS8232^ to below the limit of counting from 24 h onwards, following both the first inoculation (4 log_10_ cfu/mL) and second inoculation (5 log_10_ cfu/mL) (Figure 1e and f). No counts were detected following plating of the whole cartridge volume at 144 h after the first and second inoculations, which indicated eradication of M18^MGAS8232^.

Concentrations of 2× and 6.25× the broth microdilution MIC suppressed counts of M75^611024^ and M1T1^5448^ to below the limit of counting from 6 h onwards following both the first and second inoculations (Figure 1a–d). Concentrations of 1.5×, 2× and 6.25× the broth microdilution MIC suppressed counts of M18^MGAS8232^ to below the limit of counting from 24 h onwards for the first and second inoculations (Figure 1e and f). Plating of the whole cartridge volumes indicated eradication at concentrations of 1.5× or 2× MIC and above for both inocula and all three strains. The results following the two different inocula were very similar to each other, for all strains and penicillin G concentrations, thus practically serving as two biological replicates.

### Mathematical modelling

**Figure 2.**
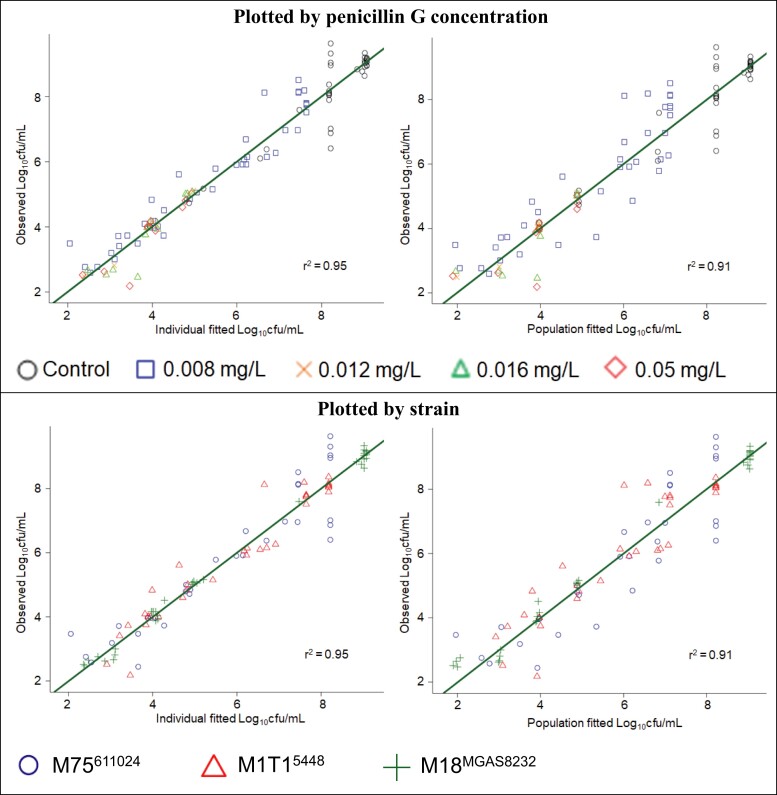
Observed versus individual fitted and population fitted viable counts, shown by penicillin G concentration and strain, in the HFIM. This figure appears in colour in the online version of *JAC* and in black and white in the print version of *JAC*.

The MBM well described the bacterial counts of all three isolates, as demonstrated by the population fitted curves, which importantly do not allow any random variability between curves (Figure 1). The individual and population fits were unbiased and sufficiently precise (Figure 2). All parameter estimates are listed in Table 1. The parameters were estimated with good precision, as the standard errors for all parameters were below 30% coefficient of variation, except Log_10,LS_ following the second inoculation of cartridges that had regrowth after the first inoculation.

The same model structure could be applied to all strains, except for the absence of a bacterial subpopulation growing at the MIC for M18^MGAS8232^. All strains could be described by the same mean generation time, maximum inhibition of successful replication (Imax_REP_) and penicillin G concentration causing 50% of Imax_REP_ for the highly susceptible subpopulation (i.e. the vast majority of the total bacterial population). M75^611024^ and M1T1^5448^ had the same maximum population size, while M18^MGAS8232^ grew to a higher maximum bacterial concentration. The slightly greater effect of 0.008 mg/L penicillin G on M75^611024^ compared with M1T1^5448^ over the first 4 days following the first inoculation could be described in the model by a lower proportion of M75^611024^ bacteria growing at the MIC (Log_10, LS_) in the initial inoculum.

## Discussion

This study involved a novel use of the HFIM to simulate a scenario of secondary prophylaxis of ARF and RHD. Therefore, relatively low bacterial inocula and penicillin G concentrations were used.^17^ For penicillin G, and β-lactams in general, the pharmacokinetic/pharmacodynamic index considered most predictive of antibacterial activity against an established infection is the duration of the dosing interval over which the unbound concentration remains above the MIC of the infecting pathogen (*fT*_>MIC_).^34–37^ In the present HFIM study, we found that concentrations at 1× the broth microdilution MIC of 0.008 mg/L decreased the bacterial counts below the limit of counting by 24 h, suppressed regrowth and achieved eradication over 6 days for strain M18^MGAS8232^, while 2× MIC (0.016 mg/L) was required to achieve the same effect on M75^611024^ and M1T1^5448^. These HFIM results align with the observation from the MBC assay that M18^MGAS8232^ counts were <1.0 log_10_ cfu/mL in the wells containing penicillin G at 1× MIC, whereas ∼2 log_10_ cfu/mL of M75^611024^ and M1T1^5448^ were still present at 1× MIC. Thus, while Strep A strains are considered universally susceptible to penicillin G,^25^ our data suggest that for some strains a small proportion (<0.1%) of the total bacterial population can survive (and subsequently grow) at 1× MIC, which may affect antibacterial outcome when bacteria are exposed to that concentration. This could explain the different HFIM results among strains with the same MIC and MBC.

By accounting for the presence of this small bacterial subpopulation (cfu_LS_) in two strains, and its absence in the third, our mathematical model could describe the bacterial counts over time for all strains simultaneously. The model incorporated the effect of penicillin G on Strep A as inhibition of successful replication (i.e. in the model bacteria that do not replicate successfully will die), in agreement with its known mechanism of action.^38^ The penicillin G concentration required for a half-maximal effect on the cfu_LS_ was estimated at 0.0094 mg/L, only slightly above the MIC. A previous mathematical model based on 24 h SCTK data from one Strep A strain used a different model structure and estimated that 0.0044 mg/L penicillin G was required for a half-maximal effect,^39^ which is within the range of the IC_50, REP_ estimates (for cfu_HS_ and cfu_LS_) from the current study. In our current model, the only other parameter estimate (apart from the proportion of bacteria growing at the MIC) that differed between strains was the maximum population size, as overall the observed plateau of the growth control was higher for M18^MGAS8232^.

There was no difference in antibacterial effect between inocula of 4 and 5 log_10_ cfu/mL for any of the strains. M75^611024^ and M1T1^5448^, which displayed regrowth at 1× MIC during the first inoculation, had an inoculum of ∼6 log_10_ cfu/mL at the start of the second inoculation; this included remaining bacteria that grew at 1× MIC during the first inoculation. Therefore, the very limited effect of penicillin G at 1× MIC on these strains following the second inoculation was likely at least in part caused by a higher proportion of bacteria in the inoculum growing at the MIC compared with the first inoculation. By including such a higher proportion following the second inoculation, the mathematical model could describe the curves starting at 6 log_10_ cfu/mL sufficiently well overall (except for underpredicting the M1T1^5448^ counts at 6 and 24 h), notably even in the population fits that do not allow for between-curve variability. An additional contributor to the attenuated effect of 1× MIC on M75^611024^ and M1T1^5448^ may have been a general inoculum effect. Very recently a 48 h HFIM study indicated an inoculum effect of a Strep A strain exposed to penicillin G.^40^ However, that study simulated an established severe acute infection and therefore examined much higher inocula (7.2 and 9.0 log_10_ cfu/mL) and penicillin G concentration (20 mg/L) compared with the current study; thus the conclusions are not directly transferable.

A systemic concentration of 0.02 mg/L penicillin G is commonly assumed to be protective against growth of Strep A for secondary prophylaxis of ARF and RHD.^12,14–16^ Assuming an unbound fraction of 0.4 in human plasma,^12,40–43^ a total concentration of 0.02 mg/L translates to an unbound concentration of 0.008 mg/L, which in the present study inhibited one strain, but not two others. The current study suggests that an unbound concentration >0.008 mg/L might provide greater protection against breakthrough of less-susceptible subpopulations in secondary prophylaxis of ARF and RHD.

In the absence of suitable human and animal models, the current study was conducted in the HFIM. The strengths of the study include the following: to the best of our knowledge, it is the first to examine the activity of penicillin G against multiple Strep A strains in the HFIM under conditions simulating secondary prophylaxis of ARF and RHD; simulation in the HFIM using experimental conditions to prevent thermal degradation of the β-lactam enabled accurate achievement of targeted, sustained penicillin G concentrations over two sequential 6 day periods, which is not possible in SCTK studies; the effects on different inocula of the isolates were examined; and, to the best of our knowledge, it is the first to develop a mathematical model for the effect of penicillin G on multiple strains of Strep A in an HFIM. The study also has some limitations. As with most *in vitro* infection models, the bacterial growth in the HFIM may not fully recapitulate that in humans. However, while erythrocyte lysate was found to inhibit the *in vitro* activity of penicillin G against *Staphylococcus aureus*, the extent of inhibition was very similar to that occurring in plasma at the same concentration of protein.^44^ Also, like other *in vitro* models, the HFIM lacks an immune system and therefore the responses observed reflect the effects of the antibiotic only. While three strains were evaluated in the current study, future experiments with additional strains may be beneficial. Nevertheless, the study provides guidance on the target concentrations of penicillin G to aim for in developing new long-acting formulations of BPG.

In conclusion, this study has provided evidence that bacterial outcomes can differ between Strep A strains even with the same MIC and MBC. Although Strep A strains are considered universally susceptible to penicillin G, the presence of a small bacterial subpopulation that grows at 1× MIC could explain the differences in outcomes between the strains, as well as largely the differences between inocula. In the current absence of validated animal and human challenge models, this HFIM study provides guidance on the target concentrations of penicillin G to aim for in ongoing efforts to develop new long-acting formulations of BPG.
